# Rosmarinic acid inhibits proliferation and migration, promotes apoptosis and enhances cisplatin sensitivity of melanoma cells through inhibiting ADAM17/EGFR/AKT/GSK3β axis

**DOI:** 10.1080/21655979.2021.1941699

**Published:** 2021-07-05

**Authors:** Lin Huang, Jiangyan Chen, Jin Quan, Debing Xiang

**Affiliations:** a Department of Dermatology, Jiangjin Central Hospital of Chongqing, Chongqing, China; b Department of Oncology, Jiangjin Central Hospital of Chongqing, Chongqing, China

**Keywords:** Rosmarinic acid, melanoma, cisplatin, ADAM17

## Abstract

Rosmarinic acid (RA), a naturally occurring polyphenolic compound, exerts multiple biological properties including anti-cancer. The metalloprotease, a disintegrin and metalloproteinase 17 (ADAM17), can activate ligands of the epidermal growth factor receptor (EGFR) and contribute to tumor progression. We aimed to investigate whether RA could exhibit anti-cancer effects in melanoma cells through down-regulating ADAM17. The human melanoma A375 cells were exposed to RA, then cell viability, migration, invasion, apoptosis, melanin content and the expression of ADAM17/EGFR/AKT/GSK3β were evaluated. The viability of cells exposed to RA in the presence of cisplatin (Cis) was measured by CCK-8. Cells were overexpressed with ADAM17 in the absence or presence of RA and ADAM17 inhibitor (TACE prodomain; TPD) co-treatment, then the above cellular processes were also observed. Results showed that A375 cells treated with RA showed significant lower cell viability, proliferation, migrative and invasive abilities, melanin content and expression of related proteins including MMP2 and MMP9, compared with normal cells. RA enhanced the ratio of TUINEL-positive cells, the expression of pro-apoptotic proteins, but reduced Bcl-2 expression. RA co-treatment increased the inhibitory effect of Cis on cell viability. RA inhibited the expression of ADAM17/EGFR/AKT/GSK3β, which was further suppressed by TPD. Moreover, ADAM17 overexpression blocked all the effects of RA whereas TPD treatment generated an opposite function. In conclusion, RA exerted obvious inhibitory effect on melanoma cell proliferation, migration and invasion, but promotive effect on cells apoptosis. Addition, the showing of this characteristic of RA may rely on inhibiting the expression of ADAM17/EGFR/AKT/GSK3β axis.

## Graphical abstract

**Figure:**
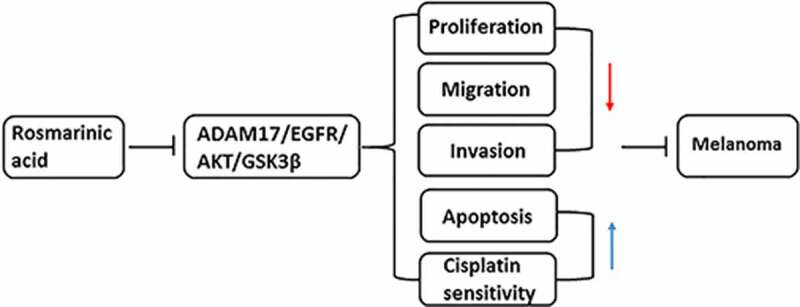

## Introduction

Malignant melanoma (MM) mostly occurs in the skin, and can also occur in the throat, nasal cavity, central nervous system, anorectum, and lymph nodes. It is a highly malignant tumor and has the highest fatality rate among skin cancers [1]. With the advancement of science and technology, the therapeutic methods for MM are diversified, including surgical treatment, radiation therapy, endocrine therapy, and immunotherapy [2]. However, because the disease is prone to hematological and lymphatic metastasis, the patient’s life expectancy is shorter, and the 5-year survival rate does not exceed 10% [3]. Therefore, in-depth exploration of the potential mechanism of MM and the search for novel specific methods and targeted drugs are currently the focus of prevention and treatment of MM.

Rosmarinic acid (RA), an ester of caffeic acid and 3,4-dihydroxyphenyllactic acidis, is a naturally water-soluble polyphenolic hydroxyl compound, and widely distributed in plants such as Lamiaceae, Boraginaceae, and Cucurbitaceae [4]. It has been confirmed that RA has multiple pharmacological effects such as anti-bacterial, antioxidant, antiviral, anti-inflammatory, anti-cancer, immune regulation, and health-enhancing activities [5–7]. RA shows great therapeutic potential in many cancers, but the anti-cancer effect of it on melanoma remains to be illustrated [5]. A previous study found that RA induced D melanogenesis through activating protein kinase A signaling [8]. Another study reported the radiosensitizing effect of RA in metastatic melanoma B16F10 cells [9]. In the present study, we aimed to investigate whether RA could exert the effects of anti-proliferation and enhancing cisplatin sensitivity in MM cells.

The metalloprotease a disintegrin and metalloproteinase 17 (ADAM17) is a member of the ADAM family of proteases, which consist of 21 members, among which 13 are active enzymes [10]. Since its discovery, ADAM17 has been implicated in the initiation and progression of practically all tumor entities, such as colon cancer, breast cancer, lung cancer and gastric cancer [11]. ADAM family have been implicated in the ectodomain shedding of six out of the seven currently known epidermal growth factor receptor (EGFR) ligands. Specifically, ADAM17 is known to have a major role in activating most ligands of EGFR, such as amphiregulin, heparin-binding epidermal growth factor and epigen, representing a crucial pathway for tumor progression [12]. In colorectal cancer, the downregulation of ADAM17 could increase the sensitivity to chemotherapy, inhibit cell proliferation, induce apoptosis, and reverse oxaliplatin resistance via suppression of the EGFR/PI3K/AKT signaling pathway [13]. Notably, RA was reported to inhibit head and neck squamous cell carcinoma in vitro through a reduction in EGFR activation [14]. ADAM17 is highly expressed in nearly all cancers and is also upregulated in melanoma (https://portals.broadinstitute.org/ccle/page?gene=ADAM17). Therefore, we hypothesized that RA could down-regulate ADAM17 expression and inhibit EGFR/AKT signaling, thereby affecting the proliferation, migration and chemotherapy sensitivity of melanoma cells.

In the present study, we aimed to investigate the role of RA in the proliferation, migration, invasion, apoptosis and cisplatin sensitivity of human melanoma cells. Additionally, whether the effects of RA exerts in the progression of melanoma is related to EGFR/AKT signaling was explored.

## Materials and methods

### Cell culture and treatment

The human melanoma cell-line A375 was purchased from Cell Bank of Chinese Academy of Sciences (Shanghai, China) and cultured in DMEM medium (Thermo Fisher Scientific, USA) supplemented with 10% FBS (Gibco, USA) and 1% penicillin-streptomycin (Beyotime Institute of Biotechnology, China) in a 5% CO_2_ incubator at 37°C.

For RA treatment (purity>98%, Sigma, USA), cells that attached to the plate bottom were exposed to different concentrations of RA (0, 50, 100, and 200 μg/ml) for 24, 48 or 72 h according to a previous study [15]. For cis-platinum (Cis, purity≥98.5%, Solarbio, China) and RA co-treatment, cells were pre-treated to RA for 24 h, followed by 8 μM Cis co-treatment for another 24 h. For RA and recombinant TACE prodomain (TPD; ADAM17 inhibitor) co-treatment, cells were co-treated with 200 μg/ml RA and 1 μM E01-GS-TPD for 48 h.

### Cell transfection

Full-length cDNAs of human ADAM17 were cloned into the pcDNA3.1 vector (Thermo Fisher Scientific, Inc.). A pcDNA3.1 empty vector was used as a negative control. A375 cells were transfected with 10 mg/l pcDNA3.1-ADAM17 (ov-ADAM17) or pcDNA3.1 (ov-NC) using Lipofectamine® 2000 reagent (Invitrogen; Thermo Fisher Scientific, Inc.) according to the manufacturer’s protocol. Following 48 h of transfection, the cells were collected for subsequent experiments.

### Cell counting kit-8 (CCK-8) assay

For assessment of cell viability and cell proliferation, cells were seeded on 96-well plate and then were transfected with or without indicated plasmids in the absence or presence of RA, Cis or E01-GS-TPD. After that, these cells were analyzed using CCK-8 (Solarbio, China). Briefly, after treatment with indicated compounds, 10 μL CCK-8 working solution was added to each well, followed by being incubated with normal cell culture medium for 2 h. Finally, the optical density (OD) was evaluated at the 450 nm wavelength using a microplate reader.

### Wound healing assay

For the wound healing assay, cells were seeded on 6-well plates at the density of 5 × 10^5^ cells/well. A pipette tip was used to create a wound after treatment with indicated compounds. The cells were then cultured in serum-free medium. The migrated cells were observed at 0 and 72 h following the creation of the wound, using an inverted microscopy (Olympus Corporation).

### Transwell assay

Transwell assay was performed using 24-well culture plates with 8-mm pore inserts (Transwell; Falcon, BD Biosciences). The lower chamber was filled with 600 µL DMEM containing 10% FBS. A375 cells (1 × 10^5^ cell/well) were seeded into the upper chamber. After 24 h of incubation, the migrated cells were fixed with 5% glutaraldehyde and then stained with crystal violet, and the number of cells in the bottom well was counted by a counting chamber. Images were captured using an inverted light microscope (magnification, x100; Olympus Corporation)

### TUNEL staining

Cell apoptosis was detected using a TUNEL Assay kit (Beyotime) according to the manufacturer’s protocol. In brief, cells were fixed with 4% paraformaldehyde for 30 min, treated with PBS containing 0.3% Triton X-100 and incubated at room temperature for 5 min. After adding 50 μL TUNEL detection solution to the sample and incubating it at 37°C for 60 min in the dark, cells were washed with PBS. Apoptotic cells were observed under a fluorescence microscope (Olympus Corporation) after mounting with an anti-fluorescence quenching mounting solution.

### Melanin content measurement

Cellular melanin content was measured as previously described [16]. After treatment with indicated compounds, cells were harvested and washed twice with PBS. Then cells were lysed in 500 μL of 1 M NaOH for 1 h at 80°C, the lysates were centrifuged at 3000 g for 10 min and the absorbance was measured at 405 nm.

### Western blot analysis

Total proteins in A375 cells were extracted using RIPA lysis buffer (Beyotime) and then quantified by a BCA kit (Thermo Fisher Scientific, Inc). Equal amounts (40 μg) of each sample were separated by 8–12% SDS-PAGE, followed by being transferred onto PVDF membranes (Bio-Rad). After being blocked with 5% nonfat milk at 37°C for 2 h, the membranes were incubated with the following primary antibodies (Abcam) overnight at 4°C: Anti-MMP2 (cat. no. ab92536; 1:5000), anti-MMP9 (cat. no. ab76003; 1:10000), anti-Bcl2 (cat. no. ab32124; 1:2000), anti-Bax (cat. no. ab32503; 1:2000), anti-cleaved caspase-3 (cat. no. ab2302; 1:1000), anti-caspase-3 (cat. no. ab13847; 1:500), anti-ADAM17 (cat. no. ab57484; 1:1000), anti-EGFR (cat. no. ab52894; 1:5000), anti-Akt (cat. no. ab8805; 1:500), anti-phosphorylated (p)-Akt (cat. no. ab81283; 1:5000), anti-GSK3β (cat. no. ab32391; 1:10000), anti-p-GSK3β (cat. no. ab75841; 1:20000), and anti-GAPDH (cat. no. ab8245; 1:10000). Finally, the membranes were incubated with a horseradish-conjugated secondary antibody (goat anti-rabbit IgG; cat. no. ab205718; 1:10,000) at room temperature for 2 h and visualized by an electrochemiluminescence system (Amersham Imager 600; GE Healthcare). Image J software (version 1.52 v, National Institutes of Health) was used for densitometric analysis of western blot. The gray value of the target protein was normalized to that of GAPDH.

### Real time quantitative PCR

The total cellular RNA from A375 cells was extracted using a commercial Trizol kit (Thermo Fisher). RNA quality was determined via the A260/A280 ratio and 1.5% agarose gel electrophoresis. The cDNA was synthesized using a reverse transcriptase (Vazyme Biotech Co., Ltd.) according to the manufacturer’s instructions. A reverse-transcription system (Promega Corporation, USA) was used to RT-PCR. 5 μg of the total RNA was placed in a 20 μL reaction tube with the following primers: ADAM17, forward: 5ʹ-GGGCAGAGGGGAAGAGAGTA-3ʹ, reverse: 5ʹ-TGTGGAGACTTGAGAATGCGA-3ʹ; GAPDH, forward: 5ʹ-GAAAGCCTGCCGGTGACTAA-3ʹ, reverse: 5ʹ-TTCCCGTTCTCAGCCTTGAC-3ʹ.

Each run consisted of an initial incubation for activation of the hot-start DNA polymerase at 95°C for 5 min. The follow-up incubation was conducted on 45 cycles of denaturation at 95°C for 10 s, annealing at 58°C for 30 s and polymerizing at 60°C for 30 s. The relative levels were quantitatively analyzed using the 2^−ΔΔCq^ method [17]. The results were expressed as the ratio of the intensity of each band to the intensity of the GAPH band.

### Statistical analysis

Data were expressed as the mean ± standard deviation from at least three independent experiments. Comparisons among multiple groups were analyzed using one-way ANOVA followed by Tukey’s post hoc test. Statistical analyses were performed using GraphPad Prism software (version 6.0; GraphPad Software, Inc.). P < 0.05 was considered to indicate a statistically significant difference.

## Results

### RA inhibits proliferation, migration and invasion of melanoma cells

**Figure 1.**
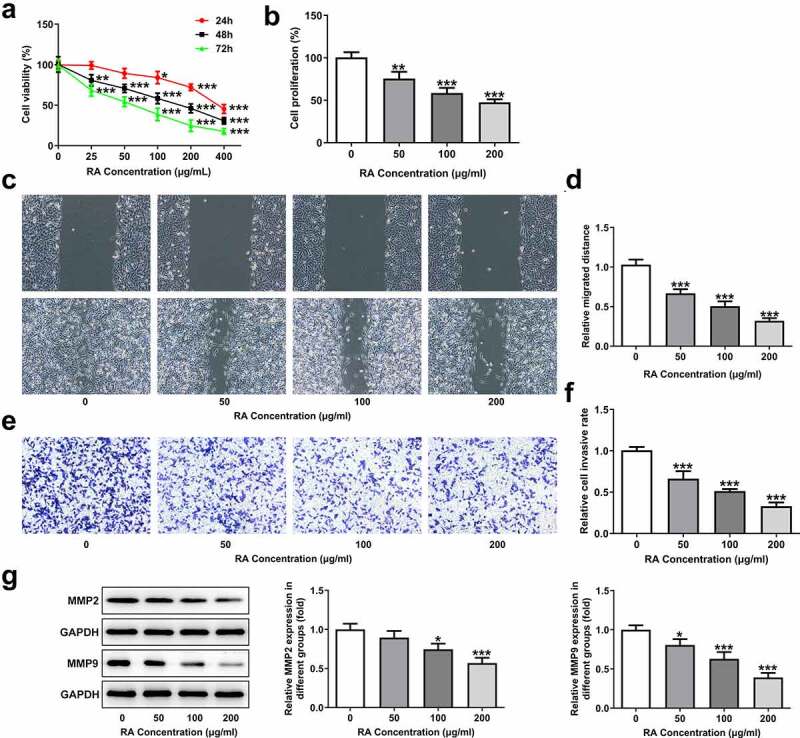
Effects of RA on proliferation, migration and invasion of melanoma cells. (a), A375 cells were exposed to different concentrations of RA for 24, 48 and 72 h, then cell viability was measured by CCK-8 assay. B-G, A375 cells were exposed to 0, 50, 100 and 200 μg/ml RA for 48 h, then cell proliferation was measured by CCK-8 assay (b); cell migration was detected via wound healing assay (c and d); cell invasion was detected via transwell assay (e and f); the protein expression of MMP2 and MMP9 was detected by western blot (g). *P < 0.05, **P < 0.01 and ***P < 0.001 vs 0 group

RA shows great therapeutic potential in many cancers, but the anti-cancer effect of it on melanoma remains to be illustrated [5]. First of all, different concentrations of RA were utilized to treat human melanoma cells A375 for 24, 48 and 72 h, and then cell viability was measured. As shown in Figure 1a, the activity of A375 cells decreased with the increase of RA concentration. At 24 h, the cell activity fell below 80% when the concentration of RA reached 200 μg/mL. At 48 h, the cell activity fell to about 50% for 200 μg/mL, and at 72 h, the cell activity fell obviously for most of concentrations. Therefore, 50, 100 and 200 μg/ml RA incubation for 48 h were selected for the following experiments. Figure 1b indicates that cell proliferation was reduced after the 48 h treatment by 50, 100 and 200 μg/ml RA. Wound healing assay was performed to observe cell migration, consequently showing that RA obviously blocked cell migration in a concentration-dependent manner (Figure 1c and d). Figure 1e demonstrates the representative images for transwell assay, and quantitative analysis in figure 1f showed that melanoma cells invasion was also prevented by RA treatment. Likewise, the expression of proteins involved in cell migration and invasion including MMP2 and MMP9 was also detected. Results in Figure 1g indicated the inhibitory effect of RA on MMP2 and MMP9 expression. The above results illustrated the repressive effect of RA on melanoma cell proliferation, migration, and invasion.

### RA promotes apoptosis, reduces melanin content and enhances Cis sensitivity of melanoma cells

**Figure 2.**
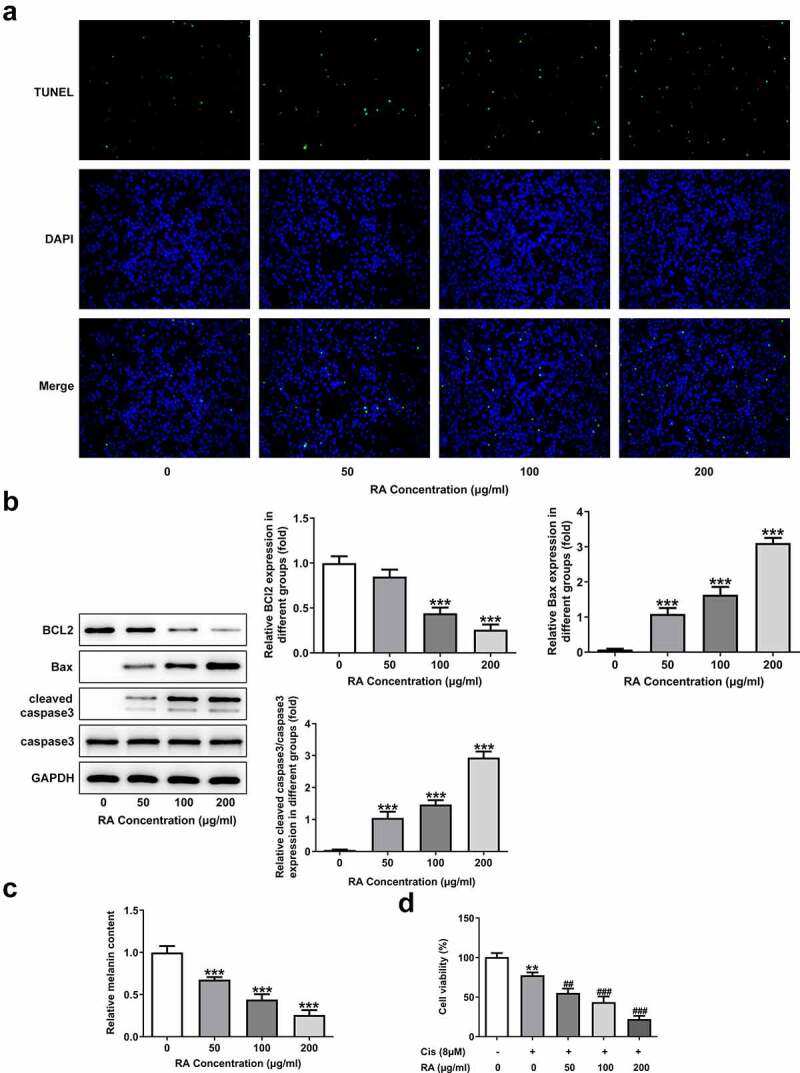
Effects of RA on apoptosis, cellular melanin content and Cis sensitivity of melanoma cells. (a-c), A375 cells were exposed to 0, 50, 100 and 200 μg/ml RA for 48 h, then cell apoptosis was observed by TUNEL staining (a); the expression of proteins involved in apoptosis including Bcl-2, Bax and cleaved-caspase3 was detected by western blot (b); the cellular melanin content was measured (c). ***P < 0.001 vs 0 group. D, A375 cells were pre-treated with 0, 50, 100 and 200 μg/ml RA for 24 h, followed by 8 μM Cis co-treatment for another 24 h, then cell viability was detected. **P < 0.01 vs 0 μg/ml RA group; ^##^P < 0.01 and ^###^P < 0.001 vs Cis + 0 μg/ml RA group

To investigate the role of RA in the apoptosis of melanoma cells, TUNEL staining was performed in our study. As shown in Figure 2a, the normal melanoma cells displayed low ratio of apoptosis (TUNEL-positive), while 50, 100 and 200 μg/ml RA treatment obviously increased the proportion of apoptotic cells. Moreover, the expression of Bcl-2 was reduced, while that of Bax and cleaved-caspase3 was increased after RA stimulation (Figure 2b). Besides, the cellular melanin content was notably reduced by 50, 100 and 200 μg/ml RA (Figure 2c). Furthermore, cell viability was also significantly inhibited by Cis and the presence of RA further enhanced this inhibitory effect, suggesting that RA could strengthen the sensitivity of melanoma cells to Cis (Figure 2d).

### RA reduces and inhibition of ADAM17 further decreases the expression of ADAM17/EGFR/AKT/GSK3β axis

**Figure 3.**
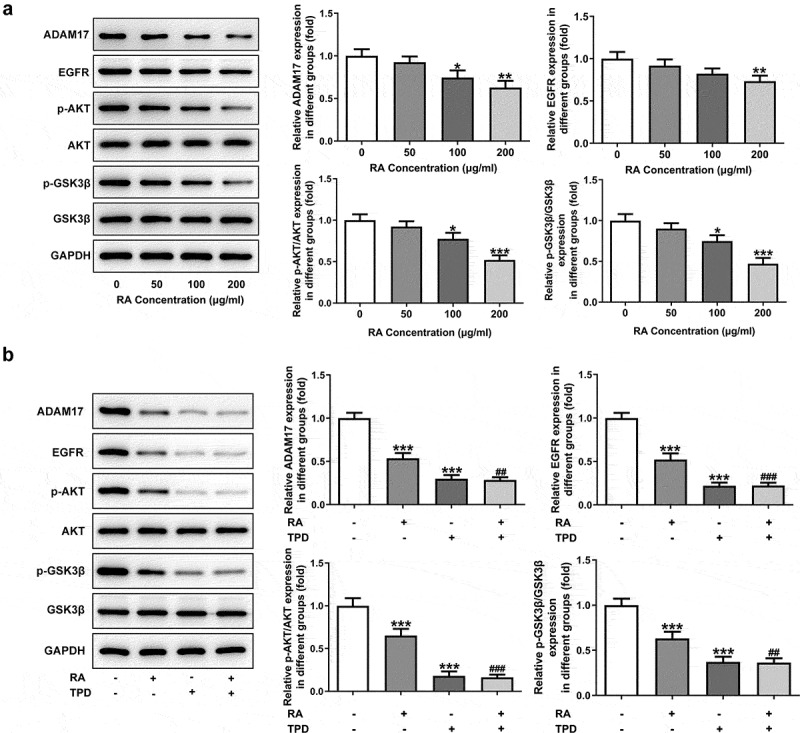
Effect of RA on the expression level of ADAM17/EGFR/AKT/GSK3β axis. (a), A375 cells were exposed to 0, 50, 100 and 200 μg/ml RA for 48 h, then the expression of proteins including ADAM17, EGFR, p-AKT and p-GSK3β was detected using western blot. *P < 0.05, **P < 0.01 and ***P < 0.001 vs 0 group. B, A375 cells were co-treated with 200 μg/ml RA and 1 μM TPD for 48 h, then the expression of proteins including ADAM17, EGFR, p-AKT and p-GSK3β was detected using western blot. ***P < 0.01 vs control group; ^##^P < 0.01 and ^###^P < 0.001 vs RA group

We next aimed to explore the possible mechanism underlying the actions of RA. As revealed in Figure 3a, the protein expression of ADAM17, EGFR, p-AKT and p-GSK3β was remarkably inhibited upon 200 μg/ml RA treatment. We then chose 200 μg/ml RA for the next research. The inhibitor of ADAM17, TPD was also used to treat cells. Similar in the function with RA, TPD also reduced the expression of ADAM17, EGFR, p-AKT and p-GSK3β (Figure 3b). Furthermore, TPD co-treatment enhanced the inhibitory effect of RA on ADAM17/EGFR/AKT/GSK3β signaling activation (Figure 3b). The above data suggested the participation of ADAM17 in the effects of RA on melanoma cells.

### ADAM17 overexpression blocks whereas TPD treatment promotes all the effects of RA on melanoma cells

**Figure 4.**
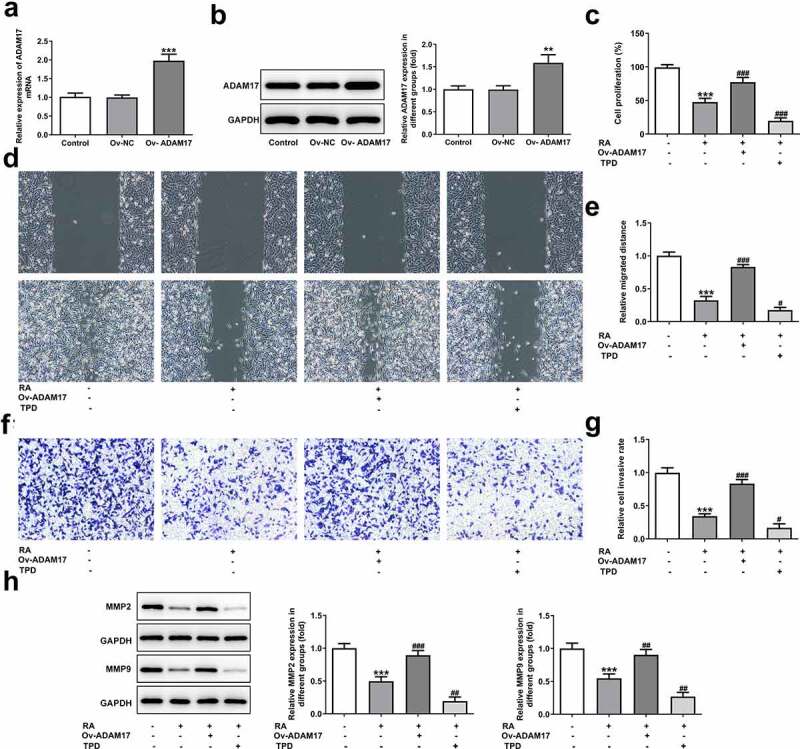
ADAM17 overexpression reverses, whereas ADAM17 inhibition enhances the effect of RA on melanoma cells proliferation, migration and invasion. A and B, A375 cells were transfected with vectors overexpressing ADAM17 or corresponding NC vectors, then mRNA (a) and protein (b) level of ADAM17 were measured. **P < 0.01 and ***P < 0.001 vs control group. C-H, A375 cells overexpressed with ADAM17 or not were exposed to RA or TPD or RA+TPD co-treatment, then cell proliferation was measured by CCK-8 assay (c); cell migration was detected via wound healing assay (d and e); cell invasion was detected via transwell assay (f and g); the protein expression of MMP2 and MMP9 was detected by western blot (h). ***P < 0.01 vs control group; ^#^P < 0.05, ^##^P < 0.01 and ^###^P < 0.001 vs RA group

To validate our hypothesis that RA could down-regulate ADAM17 expression and inhibit EGFR/AKT signaling to inhibit the progression of melanoma, ADAM17 was overexpressed in A375 cells and results in Figure 4a and Figure 4b demonstrated the significant elevation in mRNA and protein expression of ADAM17. Figure 4c shows that the cell proliferation was considerably higher in the presence of ADAM17 overexpression, but obviously lower upon TPD co-treatment in the presence of RA, compared with that of cells that were only exposed to RA. In comparison with RA treatment, overexpression of ADAM17 also increased cell migration and invasion, whereas TPD reduced these cellular abilities. Therefore, it can be concluded that ADAM17 overexpression could block whereas TPD could enhance the inhibitory effect of RA on cell migration and invasion (Figure 4d-g). The same result was also observed in Figure 4h, revealing that the expression of MMP2 and MMP9 in A375 cells, which was inhibited by RA, was significantly recovered by ADAM17 overexpression and further reduced by TPD co-treatment.

**Figure 5.**
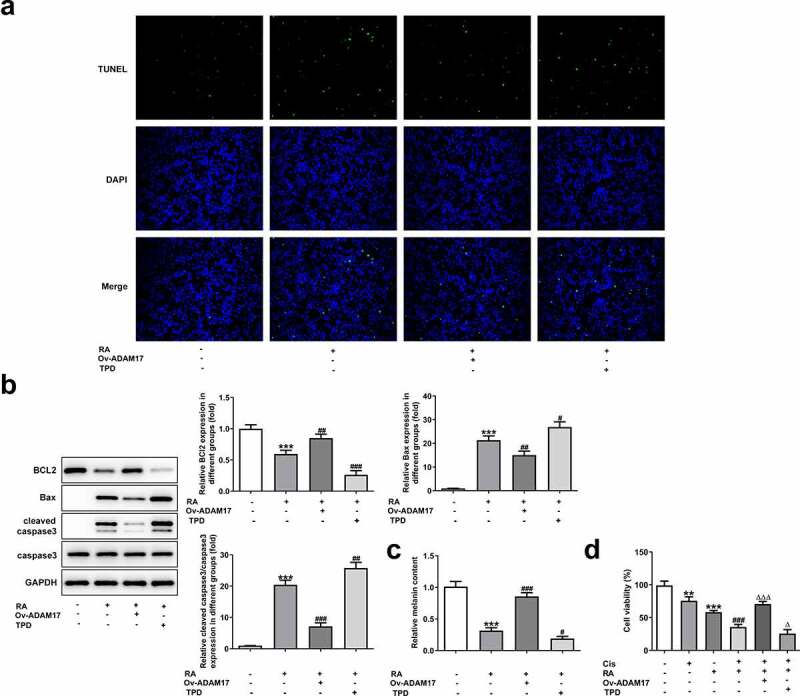
ADAM17 overexpression reverses, whereas ADAM17 inhibition enhances the effect of RA on apoptosis, cellular melanin content and Cis sensitivity of melanoma cells. (a-c), A375 cells overexpressed with ADAM17 or not were exposed to RA or TPD or RA+TPD co-treatment, then cell apoptosis was observed by TUNEL staining (a); the expression of proteins involved in apoptosis including Bcl-2, Bax and cleaved-caspase3 was detected by western blot (b); the cellular melanin content was measured (c). ***P < 0.01 vs control group; ^#^P < 0.05, ^##^P < 0.01 and ^###^P < 0.001 vs RA group. D, A375 cells were exposed to Cis (8 μM), RA (200 μg/ml), Cis + RA with or without ADAM17 overexpression, or Cis + RA + TPD, then cell viability was assessed. **P < 0.05 and ***P < 0.01 vs control group; ^###^P < 0.001 vs RA group; ^Δ^P<0.05 and ^ΔΔΔ^P<0.001 vs Cis + RA group

In addition to migration and invasion, the alteration of cell apoptosis was also observed. As shown in Figure 5 A and B, ADAM17 overexpression blunted while TPD co-treatment enhanced the promotive effect of RA on cells apoptosis. This conclusion was evidenced by decreased proportion of apoptotic cells, reduced protein expression of Bax and cleaved caspase3 together with increased Bcl-2 expression under the circumstance of ADAM17 overexpression. In comparison, the exact opposite results were revealed in the presence of TPD co-treatment, compared with RA treatment, which solidified our verdicts. In addition, ADAM17 overexpression increased whereas TPD decreased the inhibitory effect of RA on cellular melanin content (Figure 5c). Furthermore, the enhanced effect of RA on Cis sensitivity was also blunted by ADAM17 overexpression but enhanced by TPD treatment (Figure 5d).

## Discussion

In the present study, human melanoma cell-line A375 was utilized to investigate the effect of RA on melanoma and the potential mechanism. Our results revealed that RA could inhibit the cellular proliferation, migration, invasion and melanin content, but promote apoptosis and Cis sensitivity of melanoma cells in vitro. RA also down-regulated the expression level of ADAM17, EGFR, p-AKT and p-GSK3β in melanoma cells, suggesting that RA had the anti-cancer potential for melanoma treatment, and the underlying mechanism may be related to inhibiting the activation of ADAM17/EGFR/AKT/GSK3β axis. A further study was then conducted, where we used ADAM17 inhibitor-TPD to down-regulate the expression level of ADAM17 in melanoma cells. We found that the downregulation of ADAM17 not only inhibited the expression of EGFR, p-AKT, and p-GSK3β, but also further enhanced the inhibitory effect of RA on these proteins. Furthermore, we up-regulated ADAM17 in melanoma cells using overexpressing vectors and illustrated that overexpression of ADAM17 significantly blocked the effect of RA on cellular proliferation, migration, invasion, apoptosis, melanin content, and Cis sensitivity of melanoma cells. However, inhibition of ADAM17 with TPD further strengthened all the effects of RA on melanoma cells. Our results fully proved that RA played a role of protecting melanoma cells against malignant metastasis by inhibiting ADAM17/EGFR/AKT/GSK3β axis.

Plant-derived novel compounds have recently gained widespread appreciation as an alternative to traditional therapies due to their potent activity against carcinogenic cells with limited or negligible side effects [18,19]. RA is a derivative of caffeic acid and 3,4-dihydroxyphenyllactic acid that largely exists in plants such as *Rosmarinus officinalis, Perilla frutescens L., Salvia officinalis L., Mentha arvensis L*., and *Ocimum basilicum L* [20]. Current research has found that RA and its chemically derived compounds possess a wide range of pharmacological effects owing to their antitumor, anti-inflammatory, antioxidant, and antimicrobial properties [21]. Research reports in cancers have shown that RA could exert potent antitumor effects both in vitro and in vivo, through inhibiting the growth of cancer cells, and reducing the metastasis of tumors including skin cancer, pancreatic cancer, breast cancer, lung cancer, colorectal cancer and leukemia [5,22]. Studies in melanoma have shown that RA played an important role in melanogenesis and has a radio-protective impact in animals [23,24]. RA has been reported to reverse the multidrug resistance in human gastric cancer SGC7901/Adr cells [25]. RA could elevate the sensitivity of non-small cell lung cancer cells resistant to cisplatin [26]. Additionally, research has proposed that RA possesses synergistic effects with cisplatin on human ovarian cancer cells A2780 [27]. Consistent with the above reports, our results also confirmed the anti-cancer effect of RA on melanoma, as evidenced by blocked proliferation, migration, invasion, and melanin synthesis together with promotive apoptosis and Cis sensitivity in melanoma cells caused by RA.

Until now, although the potential mechanism involved in the anti-tumor effects of RA has been widely covered and multiple signaling pathways have been suggested to play a role [5,6,28], the specific mechanism related to the actions of RA in melanoma remains to be clarified. ADAM17 is highly expressed in nearly all cancers including melanoma (https://portals.broadinstitute.org/ccle/page?gene=ADAM17). Previous studies indicated that high ADAM17 gene expression correlates with poor progression-free survival in melanoma patients [29]. Additionally, ADAM17 was significantly overexpressed in advanced stage of melanoma, and the expression of TNF-α was up-regulated and significantly correlated with the expression of ADAM17 and respectively, with the advanced tumor stage [30]. Therefore, we shed light on the effect of RA on ADAM17 expression and found that RA considerably inhibited the expression of ADAM17 together with the downstream mediators of ADAM17, which constitutes ADAM17/EGFR/AKT/GSK3β axis and represents a crucial pathway for tumor progression [12]. Based on the results, we speculated that RA could target ADAM17 to down-regulate its expression therefore inactivate ADAM17/EGFR/AKT/GSK3β axis, ultimately exerting anti-cancer effects on melanoma in vitro. To further validate our hypothesis, we down-regulated and up-regulated ADAM17 expression using its inhibitor TPD, and its targeting overexpressing vectors, respectively. In accordance with our speculation, all the effects of RA on melanoma cells were significantly blocked by ADAM17 overexpression, but greatly enhanced by ADAM17 inhibition. However, this study was carried out only in A375 cells, whether the same results would be obtained in other melanoma cell lines needs to be confirmed in the subsequent experiments. Additionally, the evaluation of RA in a normal (healthy cell line) cell also get our research interest. The above two points are the limitations of the present study. Therefore, comprehensive and in-depth analysis is required in the future.

## Conclusion

Taken together, the current study for the first time shed light on the effect of RA on melanoma cells proliferation, migration, and invasion together with apoptosis and Cis sensitivity. Results illustrated that RA can inhibit the expression of ADAM17 in melanoma cells and suppress cell proliferation, invasion, migration, and melanin synthesis as well as increasing apoptosis and Cis sensitivity. This research provides a novel therapeutic approach for treating melanoma and new ideas for the mechanisms involved in the anti-tumor effects of RA.

## References

[1] Kozovska Z, Gabrisova V, Kucerova L. Malignant melanoma: diagnosis, treatment and cancer stem cells. Neoplasma. 2016;63:510–517. 27268913 10.4149/neo_2016_403

[2] Pavri SN, Clune J, Ariyan S, et al. Malignant melanoma: beyond the basics. Plast Reconstr Surg. 2016;138:330e–340e. 10.1097/PRS.0000000000002367 27465194

[3] Huang SK, Hoon DS. Liquid biopsy utility for the surveillance of cutaneous malignant melanoma patients. Mol Oncol. 2016;10:450–463. 26778792 10.1016/j.molonc.2015.12.008 PMC5307330

[4] Ngo YL, Lau CH, Chua LS. Review on rosmarinic acid extraction, fractionation and its anti-diabetic potential. Food Chem Toxicol. 2018;121:687–700. 30273632 10.1016/j.fct.2018.09.064

[5] Swamy MK, Sinniah UR, Ghasemzadeh A. Anticancer potential of rosmarinic acid and its improved production through biotechnological interventions and functional genomics. Appl Microbiol Biotechnol. 2018;102:7775–7793. 30022261 10.1007/s00253-018-9223-y

[6] Alagawany M, Abd El-Hack ME, Farag MR, et al. Rosmarinic acid: modes of action, medicinal values and health benefits. Anim Health Res Rev. 2017;18:167–176. 29110743 10.1017/S1466252317000081

[7] Rahbardar MG, Amin B, Mehri S, et al. Rosmarinic acid attenuates development and existing pain in a rat model of neuropathic pain: an evidence of anti-oxidative and anti-inflammatory effects. Phytomedicine. 2018;40:59–67. 29496176 10.1016/j.phymed.2018.01.001

[8] Lee J, Kim YS, Park D. Rosmarinic acid induces melanogenesis through protein kinase A activation signaling. Biochem Pharmacol. 2007;74(7):960–968. 17651699 10.1016/j.bcp.2007.06.007

[9] Alcaraz M, Alcaraz-Saura M, Achel DG, Lopez-Morata JA and Castillo J. Radiosensitizing effect of rosmarinic acid in metastatic melanoma B16F10 cells. Anticancer Res. 2014;34:1913–1921. 24692726

[10] Zunke F, Rose-John S. The shedding protease ADAM17: physiology and pathophysiology. Biochim biophys Acta. Mol Cell Res. 2017;1864: 2059–2070. . 28705384 10.1016/j.bbamcr.2017.07.001

[11] Düsterhöft S, Lokau J, Garbers C. The metalloprotease ADAM17 in inflammation and cancer. Pathol Res Pract. 2019;215:152410. 30992230 10.1016/j.prp.2019.04.002

[12] Moss ML, Minond D. Recent advances in ADAM17 research: a promising target for cancer and inflammation. Mediators Inflamm. 2017;2017:9673537. 29230082 10.1155/2017/9673537 PMC5688260

[13] Zhang Q, Wang C, Han X, Ge Z and Zhang G. Knockdown of ADAM17 inhibits cell proliferation and increases oxaliplatin sensitivity in HCT-8 colorectal cancer through EGFR-PI3K-AKT activation. Biochem Biophys Res Commun. 2018;503:2333–2339. 29964008 10.1016/j.bbrc.2018.06.158

[14] Waer CN, Kaur P, Tumur Z, et al. Rosmarinic acid/ blue light combination treatment inhibits head and neck squamous cell carcinoma in vitro. Anticancer Res. 2020;40:751–758. 32014917 10.21873/anticanres.14006

[15] Jang Y, Hwang K, Choi K. Rosmarinic acid, a component of rosemary tea, induced the cell cycle arrest and apoptosis through modulation of HDAC2 expression in prostate cancer cell lines. Nutrients. 2018;10. 10.3390/nu10111784 PMC6266655 30453545

[16] Chen X, Zhang B, Yuan X, et al. Isoliquiritigenin-induced differentiation in mouse melanoma B16F0 cell line. Oxid Med Cell Longev. 2012;2012:534934. 23304254 10.1155/2012/534934 PMC3529869

[17] Livak KJ, Schmittgen TD. Analysis of relative gene expression data using real-time quantitative PCR and the 2(-Delta Delta C(T)) Method. Methods. 2001;25:402–408. 11846609 10.1006/meth.2001.1262

[18] Ko YH, Kim SK, Lee SY, et al. Flavonoids as therapeutic candidates for emotional disorders such as anxiety and depression. Arch Pharm Res. 2020;43:1128–1143. 10.1007/s12272-020-01292-5 33225387

[19] Kong M, Xie K, Lv M, et al. Anti-inflammatory phytochemicals for the treatment of diabetes and its complications: lessons learned and future promise. Biomed Pharmacothe. 2020;133:110975. 10.1016/j.biopha.2020.110975 33212375

[20] Kim GD, Park YS. Jin YH and Park CS: production and applications of rosmarinic acid and structurally related compounds. Appl Microbiol Biotechnol. 2015;99:2083–2092. 25620368 10.1007/s00253-015-6395-6

[21] Petersen M, Simmonds MS. Rosmarinic acid. Phytochemistry. 2003;62:121–125. 12482446 10.1016/s0031-9422(02)00513-7

[22] Messeha SS, Zarmouh NO. Asiri A and Soliman KFA: rosmarinic acid-induced apoptosis and cell cycle arrest in triple-negative breast cancer cells. Eur J Pharmacol. 2020;885:173419. 32750370 10.1016/j.ejphar.2020.173419 PMC7541730

[23] Lin M, Zhang BX, Zhang C, et al. Ginsenosides Rb1 and Rg1 stimulate melanogenesis in human epidermal melanocytes via PKA/CREB/MITF signaling. Evid Based Complement Alternat Med. 2014;2014:892073. 24799945 10.1155/2014/892073 PMC3988736

[24] Xu W, Yang F, Zhang Y, et al. Protective effects of rosmarinic acid against radiation-induced damage to the hematopoietic system in mice. J Radiat Res. 2016;57:356–362. 27006381 10.1093/jrr/rrw021 PMC4973645

[25] Li F, Fu Y, Jiang D, et al. Reversal effect of rosmarinic acid on multidrug resistance in SGC7901/Adr cell. J Asian Nat Prod Res. 2013;15:276–285. 23421517 10.1080/10286020.2012.762910

[26] Liao XZ, Gao Y, Sun LL, et al. Rosmarinic acid reverses non-small cell lung cancer cisplatin resistance by activating the MAPK signaling pathway. Phytother Res. 2020;34:1142–1153. 31985119 10.1002/ptr.6584 PMC7217221

[27] Lešnik S, Furlan V, Bren U. Rosemary (Rosmarinus officinalis L.): extraction techniques, analytical methods and health-promoting biological effects. Phytochem Rev. 2021.

[28] Afonso AF, Pereira OR, Cardoso SM. Health-promoting effects of thymus phenolic-rich extracts: antioxidant, anti-inflammatory and antitumoral properties. Basel, Switzerland: Antioxidants;2020;9. 10.3390/antiox9090814 PMC7555682 32882987

[29] Gangemi R, Amaro A, Gino A, et al. ADAM10 correlates with uveal melanoma metastasis and promotes in vitro invasion. Pigment Cell Melanoma Res. 2014;27:1138–1148. 25124714 10.1111/pcmr.12306

[30] Cireap N, Narita D. Molecular profiling of ADAM12 and ADAM17 genes in human malignant melanoma. Pathol Oncol Res. 2013;19:755–762. 23645517 10.1007/s12253-013-9639-8

